# Redetermination of 2-[4-(2-hydroxy­ethyl)piperazin-1-ium-1-yl]ethanesul­fonate at 100 K

**DOI:** 10.1107/S1600536809042512

**Published:** 2009-11-07

**Authors:** Pawel Sledz, Thomas Minor, Maksymilian Chruszcz

**Affiliations:** a University of Virginia, Department of Molecular Physiology & Biological Physics, 1340 Jefferson Park Avenue, Charlottesville, VA 22908, USA

## Abstract

The crystal structure of the title compound (common name HEPES), C_8_H_18_N_2_O_4_S, has been redetermined at 100 K in order to properly elucidate the protonation state of the HEPES molecule. The piperazine ring has a chair conformation and one of the N atoms in the ring is protonated, which was not previously reported [Gao, Yin, Yang, & Xue (2004). *Acta Cryst.* E**60**, o1328–o1329]. The change of protonation state of the nitrogen atom significantly affects the intermolecular interactions in the HEPES crystal. The structure is stabilized by N—H⋯O and O—H⋯O hydrogen bonds and ionic inter­actions, as the title compound in solid state is a zwitterion. HEPES mol­ecules pack in layers that are held together by ionic and weak inter­actions, while a hydrogen-bonded network connects the layers.

## Related literature

For background to HEPES and analogous compounds, see: Ferguson *et al.* (1980 ▶); Good & Izawa (1972 ▶); Good *et al.* (1966 ▶). For the crystal structure of HEPES crystallized from methanol, see: Wouters *et al.* (1996 ▶) and from water, see: Gao *et al.* (2004 ▶). For related structures, see: Kubicki *et al.* (2007 ▶); Chruszcz *et al.* (2005 ▶); Zhao *et al.* (2006 ▶).
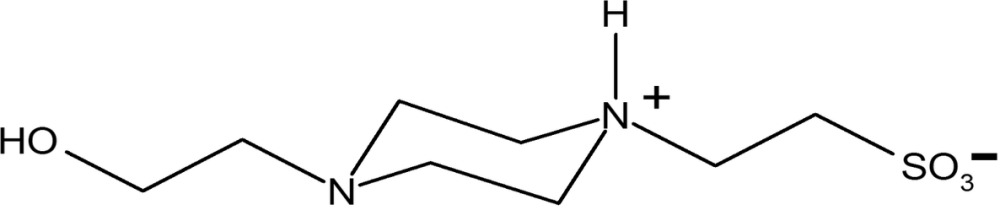


## Experimental

### 

#### Crystal data

C_8_H_18_N_2_O_4_S
*M* *_r_* = 238.31
Orthorhombic, 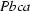

*a* = 8.341 (1) Å
*b* = 9.567 (1) Å
*c* = 27.066 (1) Å
*V* = 2159.8 (4) Å^3^
*Z* = 8
Mo *K*α radiation
μ = 0.30 mm^−1^
*T* = 100 K
0.50 × 0.50 × 0.23 mm

#### Data collection

Rigaku R-AXIS RAPID diffractometer
Absorption correction: multi-scan (Otwinowski *et al.*, 2003 ▶) *T*_min_ = 0.86, *T*_max_ = 0.93
613697 measured reflections
17694 independent reflections
14854 reflections with *I* > 2σ(*I*)
*R*_int_ = 0.036

#### Refinement

*R*[*F* ^2^ > 2σ(*F* ^2^)] = 0.031
*wR*(*F* ^2^) = 0.099
*S* = 1.04
17694 reflections
208 parameters
All H-atom parameters refined
Δρ_max_ = 0.83 e Å^−3^
Δρ_min_ = −0.80 e Å^−3^

### 

Data collection: *HKL-2000* (Otwinowski & Minor, 1997 ▶); cell refinement: *HKL-2000*; data reduction: *HKL-2000*; program(s) used to solve structure: *SHELXS97* (Sheldrick, 2008 ▶) and *HKL-3000SM* (Minor *et al.*, 2006 ▶); program(s) used to refine structure: *SHELXL97* (Sheldrick, 2008 ▶) and *HKL-3000SM*; molecular graphics: *HKL-3000SM*, *ORTEPIII* (Burnett & Johnson, 1996 ▶), *ORTEP-3* (Farrugia, 1997 ▶), *Mercury* (Macrae *et al.*, 2006 ▶) and *POV-RAY* (The *POV-RAY* Team, 2004 ▶); software used to prepare material for publication: *HKL-3000SM*.

## Supplementary Material

Crystal structure: contains datablocks I, global. DOI: 10.1107/S1600536809042512/fl2269sup1.cif

Structure factors: contains datablocks I. DOI: 10.1107/S1600536809042512/fl2269Isup2.hkl

Additional supplementary materials:  crystallographic information; 3D view; checkCIF report

## Figures and Tables

**Table 1:** Hydrogen-bond geometry (Å, °)

*D*—H⋯*A*	*D*—H	H⋯*A*	*D*⋯*A*	*D*—H⋯*A*
O4—H1*O*4⋯N2^i^	0.85 (1)	1.99 (1)	2.8368 (4)	173 (1)
N1—H1*N*⋯O2^ii^	0.83 (1)	1.92 (1)	2.7414 (4)	169 (1)

Symmetry codes: (i) 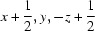; (ii) 
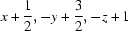
.

**Fig. 1.**
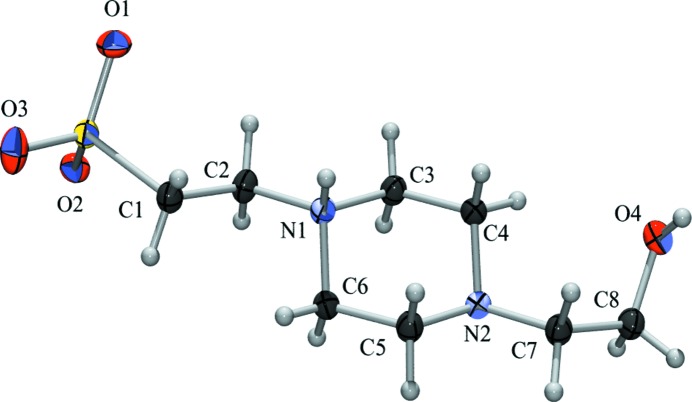
The molecular structures of the title compound. Displacement ellipsoids are drawn at the 75% probability level and hydrogen atoms are drawn as spheres of an arbitrary radius.

**Fig. 2.**
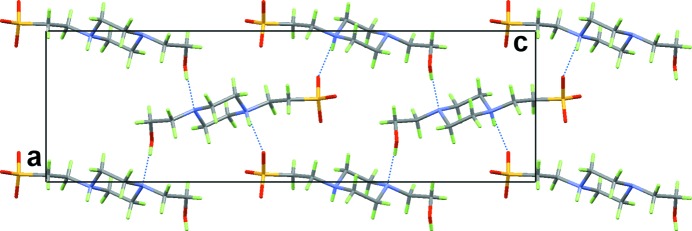
The molecular packing of compound (I) shown along the [010] axis. Hydrogen atoms are bonds are marked in green, and H-bonds are shown as blue, dashed lines.

**Fig. 3.**
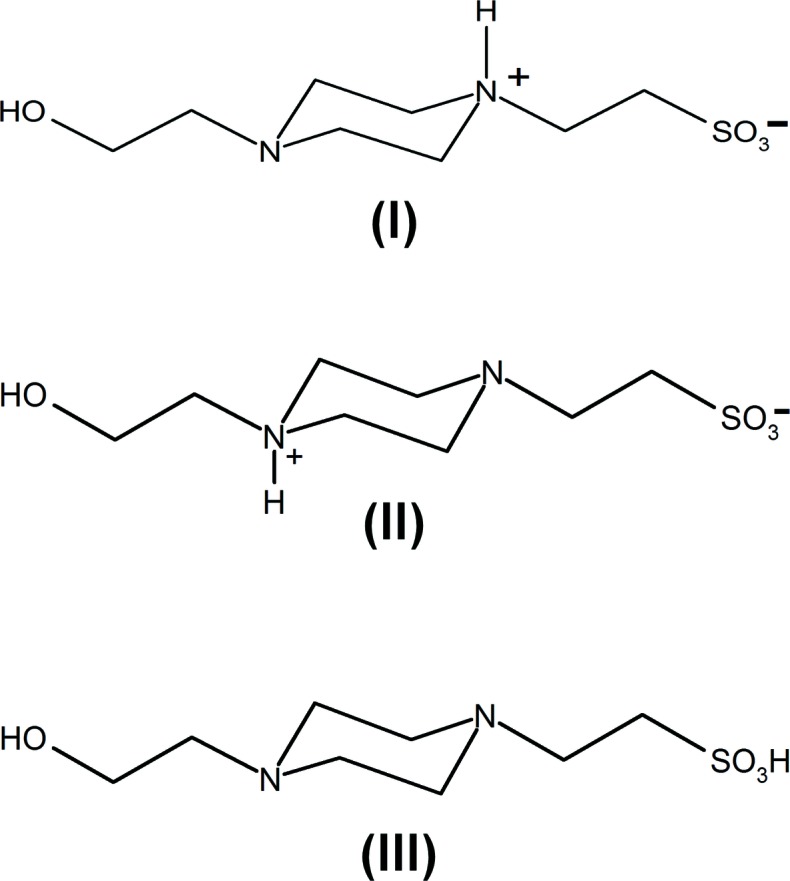
The structures of (I), (II) and (III).

## supplementary crystallographic information

### Comment

The title compound, (I), known commonly as HEPES, is a sulfonic compound used as a zwitterionic buffer. HEPES and analogous compounds (known as the Good buffers) are used during the study of biological processes (Good *et al.*, 1966; Good & Izawa, 1972; Ferguson *et al.*, 1980), and very often during crystallization of macromolecules.

The crystal structures of HEPES crystallized from methanol (determined at 293 K - Wouters *et al.*, 1996) and from water (298 K - Gao *et al.*, 2004) have been already reported. Both previously reported structures crystallized in the Pbca space group, but with different unit-cell parameters. Not only the unit-cell parameters, but also the conformations of the HEPES molecules were different. However, our attention was turned to differences in the reported protonation states of the HEPES molecules. In the structure reported by Wouters *et al.*, the HEPES molecule was presented as zwitterionic (II), while in structure reported by Gao *et al.* HEPES had both piperazine nitrogen atoms non-protonated and a protonated sulfonic group (III). The non-zwitterionic form of HEPES is quite unusual, as all previously determined structures of compounds from this group, *e.g.* MES [2-(*N*-morpholino)ethanesulfonic acid] (Kubicki *et al.*, 2007), MOPS [3-(*N*-morpholino)propanesulfonic acid] (Chruszcz *et al.*, 2005), PBHPS [piperazine-1,4-diylbis(2-hydroxypropanesulfonic acid)] (Zhao *et al.*, 2006), consistently report zwitterionic forms as being observed in the solid state.

In order to localize all hydrogen atoms, the structure determination was performed at 100 K. The crystal structure of the title compound reported here is isomorphous to the structure reported by Gao *et al.*, but a more detailed analysis revealed that the HEPES molecules are zwitterionic and the nitrogen atom (N1) is protonated (Fig. 1). Change of the localization of the hydrogen atom in comparison with the previously reported structure (Gao *et al.*, 2004) significantly affects the hydrogen bond network (Fig. 2, Table 1), which we believed was previously incorrectly determined. To confirm our finding, we also performed a structural analysis of HEPES crystals (crystallized from water or taken directly from the bottle provided by SIGMA) at 293 K. In both cases (100 K and 293 K) the structures had the same protonation state, which excluded the possibility of temperature dependent changes of protonation.

The differing protonation states reported here and in the structure determined by Wouters *et al.* suggest that the change in protonation state of the HEPES molecule (and/or the change of the conformations of 2-hydroxyethyl and enthanesulfonic moieties) results in generation of polymorphic forms.

The crystal structure of HEPES is stabilized mainly by hydrogen bonds and ionic interactions. Both nitrogen atoms from the piperazine ring, oxygen atom (O4) from the hydroxyl group and one of the oxygen atom (O2) from the sulfonic group are involved in formation of the hydrogen bond network. Hydrogen bonds extend along the [100] direction and details of their geometric parameters are summarized in Table 1.The HEPES molecules pack in layers that are held together by ionic and weak interactions.

### Experimental

HEPES was purchased from SIGMA (99.5% purity, lot 036 K5461). The crystal of (I), used for X-ray diffraction study, was obtained by slow evaporation of HEPES solution in water.

### Refinement

All hydrogen atoms were localized using the difference density Fourier map. Their positions and isotropic displacement parameters were refined.

### Crystal data

**Table:**

C_8_H_18_N_2_O_4_S	*F*(000) = 1024.0
*M**_r_* = 238.31	*D*_x_ = 1.466 Mg m^−^^3^
Orthorhombic, *P**b**c**a*	Mo *K*α radiation, λ = 0.7107 Å
Hall symbol: -P 2ac 2ab	Cell parameters from 613697 reflections
*a* = 8.341 (1) Å	θ = 2.9–62.9°
*b* = 9.567 (1) Å	µ = 0.30 mm^−^^1^
*c* = 27.066 (1) Å	*T* = 100 K
*V* = 2159.8 (4) Å^3^	Block, colorless
*Z* = 8	0.50 × 0.50 × 0.23 mm

### Data collection

**Table:**

Rigaku R-AXIS RAPID diffractometer	17694 independent reflections
Radiation source: fine-focus sealed tube	14854 reflections with *I* > 2σ(*I*)
graphite	*R*_int_ = 0.036
Detector resolution: 10 pixels mm^-1^	θ_max_ = 62.9°, θ_min_ = 2.9°
ω scan with χ offset	*h* = −20→20
Absorption correction: multi-scan (Otwinowski *et al.*, 2003)	*k* = −23→23
*T*_min_ = 0.86, *T*_max_ = 0.93	*l* = −67→67
613697 measured reflections	

### Refinement

**Table:**

Refinement on *F*^2^	Primary atom site location: structure-invariant direct methods
Least-squares matrix: full	Secondary atom site location: difference Fourier map
*R*[*F*^2^ > 2σ(*F*^2^)] = 0.031	Hydrogen site location: difference Fourier map
*wR*(*F*^2^) = 0.099	All H-atom parameters refined
*S* = 1.04	*w* = 1/[σ^2^(*F*_o_^2^) + (0.060*P*)^2^ + 0.0821*P*] where *P* = (*F*_o_^2^ + 2*F*_c_^2^)/3
17694 reflections	(Δ/σ)_max_ = 0.013
208 parameters	Δρ_max_ = 0.83 e Å^−^^3^
0 restraints	Δρ_min_ = −0.80 e Å^−^^3^

### Special details

**Table:**

Geometry. All e.s.d.'s (except the e.s.d. in the dihedral angle between two l.s. planes) are estimated using the full covariance matrix. The cell e.s.d.'s are taken into account individually in the estimation of e.s.d.'s in distances, angles and torsion angles; correlations between e.s.d.'s in cell parameters are only used when they are defined by crystal symmetry. An approximate (isotropic) treatment of cell e.s.d.'s is used for estimating e.s.d.'s involving l.s. planes.
Refinement. Refinement of *F*^2^ against ALL reflections. The weighted *R*-factor *wR* and goodness of fit *S* are based on *F*^2^, conventional *R*-factors *R* are based on *F*, with *F* set to zero for negative *F*^2^. The threshold expression of *F*^2^ > σ(*F*^2^) is used only for calculating *R*-factors(gt) *etc*. and is not relevant to the choice of reflections for refinement. *R*-factors based on *F*^2^ are statistically about twice as large as those based on *F*, and *R*- factors based on ALL data will be even larger.

### Fractional atomic coordinates and isotropic or equivalent isotropic displacement parameters (Å^2^)

**Table:**

	*x*	*y*	*z*	*U*_iso_*/*U*_eq_	
S1	0.449764 (8)	0.776637 (6)	0.555875 (2)	0.01145 (1)	
N2	0.52317 (2)	0.69925 (2)	0.302785 (7)	0.01111 (2)	
O2	0.30713 (2)	0.86815 (2)	0.555768 (7)	0.01413 (3)	
N1	0.53191 (2)	0.75196 (2)	0.408697 (7)	0.01121 (2)	
O4	0.72742 (3)	0.83527 (2)	0.216353 (8)	0.01514 (3)	
C1	0.45506 (3)	0.69919 (3)	0.495798 (9)	0.01321 (3)	
C5	0.49778 (3)	0.58710 (2)	0.339211 (8)	0.01316 (3)	
O1	0.59735 (3)	0.85596 (3)	0.560910 (9)	0.01744 (3)	
C3	0.56026 (3)	0.86534 (2)	0.371519 (9)	0.01330 (3)	
C6	0.42540 (3)	0.64240 (2)	0.386736 (8)	0.01298 (3)	
C4	0.63131 (3)	0.80396 (2)	0.324618 (8)	0.01271 (3)	
C2	0.46765 (3)	0.81142 (3)	0.456032 (9)	0.01364 (3)	
C8	0.59941 (3)	0.73733 (3)	0.213505 (9)	0.01485 (3)	
C7	0.58957 (3)	0.63743 (2)	0.257306 (8)	0.01343 (3)	
O3	0.43278 (4)	0.66097 (3)	0.590283 (9)	0.02055 (4)	
H1A	0.5416 (10)	0.6363 (10)	0.4955 (4)	0.030 (2)*	
H7B	0.5234 (9)	0.5552 (9)	0.2488 (3)	0.0238 (17)*	
H2A	0.5445 (9)	0.8874 (10)	0.4673 (3)	0.0253 (19)*	
H4B	0.6453 (10)	0.8784 (8)	0.3001 (3)	0.0252 (16)*	
H7A	0.7005 (8)	0.5925 (7)	0.2643 (3)	0.0184 (14)*	
H3B	0.4576 (9)	0.9063 (10)	0.3665 (3)	0.0253 (19)*	
H6A	0.4173 (11)	0.5721 (9)	0.4086 (3)	0.030 (2)*	
H6B	0.3201 (9)	0.6878 (8)	0.3822 (3)	0.0199 (15)*	
H4A	0.7367 (10)	0.7630 (8)	0.3328 (3)	0.0193 (15)*	
H1O4	0.8138 (12)	0.7897 (9)	0.2127 (3)	0.038 (2)*	
H5A	0.6006 (9)	0.5394 (8)	0.3472 (3)	0.0175 (14)*	
H3A	0.6309 (10)	0.9315 (7)	0.3860 (3)	0.0217 (16)*	
H5B	0.4242 (10)	0.5192 (9)	0.3260 (3)	0.0249 (18)*	
H1B	0.3616 (9)	0.6429 (8)	0.4933 (3)	0.0220 (16)*	
H8B	0.5045 (10)	0.7906 (9)	0.2102 (3)	0.0225 (17)*	
H1N	0.6197 (10)	0.7168 (7)	0.4156 (3)	0.0209 (16)*	
H2B	0.3673 (10)	0.8534 (8)	0.4489 (3)	0.0235 (17)*	
H8A	0.6076 (9)	0.6844 (8)	0.1846 (3)	0.0193 (15)*	

### Atomic displacement parameters (Å^2^)

**Table:**

	*U*^11^	*U*^22^	*U*^33^	*U*^12^	*U*^13^	*U*^23^
S1	0.01147 (2)	0.01296 (2)	0.00992 (2)	0.00039 (1)	0.00046 (1)	0.00017 (1)
N2	0.01141 (5)	0.01171 (5)	0.01023 (5)	−0.00043 (4)	0.00008 (4)	−0.00049 (4)
O2	0.01144 (6)	0.01507 (5)	0.01589 (6)	0.00088 (4)	0.00135 (4)	−0.00176 (4)
N1	0.01141 (5)	0.01222 (5)	0.01001 (5)	0.00009 (4)	0.00001 (4)	−0.00027 (4)
O4	0.01385 (6)	0.01526 (6)	0.01631 (6)	0.00036 (5)	0.00216 (5)	0.00128 (4)
C1	0.01505 (8)	0.01313 (6)	0.01144 (6)	−0.00002 (5)	0.00161 (5)	−0.00042 (5)
C5	0.01598 (8)	0.01164 (6)	0.01185 (6)	−0.00126 (5)	0.00163 (5)	−0.00039 (4)
O1	0.01166 (6)	0.02271 (8)	0.01794 (7)	−0.00175 (5)	−0.00219 (5)	−0.00297 (6)
C3	0.01666 (8)	0.01192 (6)	0.01131 (6)	−0.00133 (5)	0.00028 (5)	−0.00032 (4)
C6	0.01328 (7)	0.01395 (6)	0.01170 (6)	−0.00217 (5)	0.00141 (5)	−0.00079 (5)
C4	0.01330 (7)	0.01364 (6)	0.01120 (6)	−0.00253 (5)	0.00046 (5)	−0.00035 (5)
C2	0.01686 (8)	0.01329 (6)	0.01078 (6)	0.00146 (6)	0.00107 (5)	−0.00046 (5)
C8	0.01395 (8)	0.01909 (8)	0.01151 (6)	−0.00078 (6)	0.00002 (5)	0.00110 (5)
C7	0.01524 (8)	0.01335 (6)	0.01171 (6)	−0.00001 (5)	0.00150 (5)	−0.00108 (5)
O3	0.03015 (11)	0.01750 (7)	0.01399 (6)	0.00248 (7)	0.00373 (6)	0.00455 (5)

### Geometric parameters (Å, °)

**Table:**

S1—O1	1.4525 (3)	C5—H5A	0.995 (8)
S1—O3	1.4532 (2)	C5—H5B	0.963 (8)
S1—O2	1.4771 (2)	C3—C4	1.5190 (3)
S1—C1	1.7874 (3)	C3—H3B	0.951 (8)
N2—C4	1.4719 (3)	C3—H3A	0.949 (8)
N2—C5	1.4724 (3)	C6—H6A	0.898 (9)
N2—C7	1.4736 (3)	C6—H6B	0.987 (8)
N1—C6	1.4971 (3)	C4—H4B	0.981 (8)
N1—C3	1.4984 (3)	C4—H4A	0.988 (8)
N1—C2	1.5008 (3)	C2—H2A	1.016 (9)
N1—H1N	0.827 (8)	C2—H2B	0.948 (8)
O4—C8	1.4226 (4)	C8—C7	1.5250 (4)
O4—H1O4	0.848 (10)	C8—H8B	0.946 (9)
C1—C2	1.5239 (4)	C8—H8A	0.935 (8)
C1—H1A	0.939 (9)	C7—H7B	0.988 (8)
C1—H1B	0.950 (8)	C7—H7A	1.037 (7)
C5—C6	1.5162 (3)		
			
O1—S1—O3	114.856 (17)	C4—C3—H3A	111.1 (5)
O1—S1—O2	111.907 (17)	H3B—C3—H3A	110.1 (7)
O3—S1—O2	111.966 (15)	N1—C6—C5	110.17 (2)
O1—S1—C1	106.317 (13)	N1—C6—H6A	107.9 (6)
O3—S1—C1	105.650 (15)	C5—C6—H6A	109.1 (6)
O2—S1—C1	105.296 (12)	N1—C6—H6B	105.6 (4)
C4—N2—C5	108.376 (18)	C5—C6—H6B	113.7 (4)
C4—N2—C7	112.22 (2)	H6A—C6—H6B	110.1 (7)
C5—N2—C7	108.719 (19)	N2—C4—C3	111.07 (2)
C6—N1—C3	109.504 (18)	N2—C4—H4B	107.2 (5)
C6—N1—C2	113.10 (2)	C3—C4—H4B	109.3 (5)
C3—N1—C2	110.809 (19)	N2—C4—H4A	111.4 (4)
C6—N1—H1N	109.3 (5)	C3—C4—H4A	108.3 (4)
C3—N1—H1N	107.8 (5)	H4B—C4—H4A	109.5 (6)
C2—N1—H1N	106.1 (5)	N1—C2—C1	111.129 (19)
C8—O4—H1O4	107.0 (6)	N1—C2—H2A	107.6 (5)
C2—C1—S1	110.620 (17)	C1—C2—H2A	109.6 (5)
C2—C1—H1A	113.1 (6)	N1—C2—H2B	107.6 (5)
S1—C1—H1A	106.9 (6)	C1—C2—H2B	112.4 (5)
C2—C1—H1B	113.9 (5)	H2A—C2—H2B	108.4 (7)
S1—C1—H1B	106.2 (5)	O4—C8—C7	114.27 (2)
H1A—C1—H1B	105.5 (8)	O4—C8—H8B	106.2 (5)
N2—C5—C6	111.782 (19)	C7—C8—H8B	111.5 (5)
N2—C5—H5A	110.8 (4)	O4—C8—H8A	110.3 (5)
C6—C5—H5A	108.7 (4)	C7—C8—H8A	108.4 (5)
N2—C5—H5B	109.5 (5)	H8B—C8—H8A	105.8 (7)
C6—C5—H5B	107.3 (5)	N2—C7—C8	114.71 (2)
H5A—C5—H5B	108.7 (7)	N2—C7—H7B	107.7 (5)
N1—C3—C4	110.055 (19)	C8—C7—H7B	110.3 (5)
N1—C3—H3B	104.6 (5)	N2—C7—H7A	110.5 (4)
C4—C3—H3B	113.0 (5)	C8—C7—H7A	110.7 (4)
N1—C3—H3A	107.7 (4)	H7B—C7—H7A	102.1 (5)
			
O1—S1—C1—C2	−59.26 (2)	C5—N2—C4—C3	−59.66 (2)
O3—S1—C1—C2	178.27 (2)	C7—N2—C4—C3	−179.726 (19)
O2—S1—C1—C2	59.63 (2)	N1—C3—C4—N2	59.63 (3)
C4—N2—C5—C6	59.21 (3)	C6—N1—C2—C1	62.57 (3)
C7—N2—C5—C6	−178.55 (2)	C3—N1—C2—C1	−174.03 (2)
C6—N1—C3—C4	−56.75 (3)	S1—C1—C2—N1	159.105 (18)
C2—N1—C3—C4	177.80 (2)	C4—N2—C7—C8	−68.69 (3)
C3—N1—C6—C5	55.97 (3)	C5—N2—C7—C8	171.44 (2)
C2—N1—C6—C5	−179.911 (19)	O4—C8—C7—N2	76.07 (3)
N2—C5—C6—N1	−58.33 (3)		

### Hydrogen-bond geometry (Å, °)

**Table:**

*D*—H···*A*	*D*—H	H···*A*	*D*···*A*	*D*—H···*A*
O4—H1O4···N2^i^	0.85 (1)	1.99 (1)	2.8368 (4)	173 (1)
N1—H1N···O2^ii^	0.83 (1)	1.92 (1)	2.7414 (4)	169 (1)

Symmetry codes: (i) *x*+1/2, *y*, −*z*+1/2; (ii) *x*+1/2, −*y*+3/2, −*z*+1.
